# Renoprotective impact of tiron against diclofenac-induced nephrotoxicity: targeting TLR4/NF-κB/NLRP3/Caspase-1/IL1-β pathway

**DOI:** 10.1186/s40360-025-01012-z

**Published:** 2025-10-30

**Authors:** Aya S. Ragab, Alaa S. El-Kelany, Haitham M. Sewilam, Dalia H. El-Kashef

**Affiliations:** 1 Department of Pharmacology and Toxicology, Faculty of Pharmacy, Mansoura University, Mansoura, 35516 Egypt; 2 Department of Histology, Faculty of Medicine, Helwan University, Cairo, Egypt

**Keywords:** Diclofenac, Nephrotoxicity, Tiron, Oxidative stress, Inflammation

## Abstract

Nephrotoxicity is a documented side effect of non-steroidal anti-inflammatory drugs (NSAIDs) like diclofenac (Diclo). Thus, this study was executed to assess the renoprotective effect of tiron against Diclo-induced nephrotoxicity. Nephrotoxicity was induced in mice by single administration of Diclo (300 mg/kg, po). Mice received tiron (140 and 280 mg/kg, ip) for 7 successive days, Diclo was administered on day 7 after 1 h of tiron injection. Diclo significantly deteriorated kidney function and structure; Diclo injection produced a marked increase in serum levels of creatinine, urea and blood urea nitrogen (BUN) as well as profound escalation in levels of creatinine, total protein and albumin in urine. Moreover, Diclo induced oxidative stress manifested by substantial increase in malondialdehyde (MDA) and notable decrease in reduced glutathione (GSH) level and superoxide dismutase (SOD) activity. Additionally, Diclo significantly increased renal levels of toll-like receptor 4 (TLR-4), nuclear factor kappa B (NF-κB), NOD-like receptor protein 3 (NLRP3), apoptosis associated speck-like protein, Caspase-1 and interleukin (IL)-1β, besides elevation in renal expression of cyclooxygenase (COX)-II. The results of these biomarkers were further confirmed by histopathological analysis. Injection of tiron in both doses markedly improved Diclo-induced alterations. Hence, tiron could be a promising candidate in alleviating nephrotoxicity induced by NSAIDs following further clinical research.

## Highlights

- Tiron exerted renoprotective effect against diclofenac-induced nephrotoxicity in mice via decreasing levels of serum creatinine and blood urea nitrogen.
- Tiron improved kidney architecture as evidenced by histopathological observations.
- Tiron possessed anti-oxidant effects through decreasing MDA content and increasing GSH level and SOD activity.
- Tiron decreased inflammation via down-regulating TLR4/NF-κB/NLRP3/Caspase-1/IL1-β pathway.

## Introduction

The kidney is crucial for preserving homeostasis and detoxifying a variety of endogenous compounds and hydrophilic xenobiotics. The kidney is at high risk for injury due to its exposure to the damage caused by elevated concentrations of drugs and toxins compared to other organs. This could be attributed to its role in the tubular excretion of drugs and the reabsorption of filtered toxins into the tubular lumen [1].

Nephrotoxicity occurs as a result of exogenous or endogenous toxicants damaging or destroying kidney function, which impairs kidney-specific detoxification and excretion [2]. Non-steroidal anti-inflammatory drugs (NSAIDs) are widely used for the treating different clinical conditions such as rheumatoid arthritis, fever, inflammation, acute injury pain, osteoarthritis and ischemic heart disease. Among NSAIDs, diclofenac (Diclo) is the most widely significant and extensively prescribed drug worldwide due to its analgesic, antipyretic and anti-inflammatory activities. The suggested dosages of Diclo are often both safe and efficient. However, higher dosages used for longer periods of time may cause acute kidney injury, which, if left untreated, may result in insufficient renal recovery or hasten the development of chronic kidney disease [3].

The mechanisms behind Diclo-induced nephrotoxicity are not fully understood, however there are some proposed mechanisms. One of them is the property of Diclo to inhibit prostaglandins (PGs), which has negative effects via inhibiting COX enzymes. By preventing PG production, Diclo has been shown to decrease glomerular filtration rate and other renal functions in a dose-dependent way. Additionally, Diclo can damage renal proximal tubules by increasing intracellular osmolality and triggering autolysis, which results in kidney tubular dysfunction and a decrease in glomerular filtration rate [4]. Another hypothesis is the ability of Diclo to induce oxidative stress and lipid peroxidation that result mainly from Diclo metabolism to its active metabolites [3, 5]. The production of reactive oxygen species (ROS) causes heightened inflammatory reactions by activating nuclear factor kappa B (NF-κB) transcription, which in turn causes acute kidney injury to proceed further [6].

Tiron is an analogue of vitamin E (alpha-tocopherol) that dissolves in water. It is an antioxidant and active, nontoxic metal chelating agent that can prevent oxidative damage caused by ROS and activate antioxidant enzymes [7]. Because of its small size and cell-permeable properties, it can scavenge free radicals and penetrate the mitochondria to alter intracellular electron transfer reactions [8]. Additionally, tiron has been shown to have protective effects against myocardial injury induced by isoprenaline [9]. Furthermore, its beneficial effects against parkinsonism [10] and breast cancer [7] have been reported.

In light of these factors, the current study was performed to investigate the possible protective impacts of tiron against Diclo-induced nephrotoxicity and the fundamental mechanisms behind these effects.

## Materials and methods

### Chemicals

Diclo was bought as commercial preparation (Voltaren^®^ tablets, Novartis, Egypt). Tiron was obtained as pure powder (Acros Organics, Belgium) and dissolved in normal saline.

### Animals

Thirty two male albino mice of weight 23 ± 2 g were utilized in this experiment. These mice were placed in a plastic cages at room temperature, supplied with free food and water with 12 h light/dark cycle for 7 days prior to study. Animal care and experimental procedures also adhere to the ethical guidelines approved by the “Research Ethical Committee” of Faculty of Pharmacy Mansoura University (code number: 2025-13).

### Methods

Mice were classified haphazardly into four groups (*n* = 8) as follow:

#### Control group

Mice received vehicles only.

#### Diclo group

Mice received toxic dose of Diclo (300 mg/kg, po) at day seven [11].

#### Tiron 1 group

Mice received tiron (140 mg/kg, ip) [9] for seven days then received single dose of Diclo on day 7.

#### Tiron 2 group

Mice received tiron (280 mg/kg, ip) [1] for seven days then received single dose of Diclo on day 7.

After Diclo administration, all mice were kept in metabolic cages to collect 24 h urine samples. On day 8, all mice were weighed and euthanized by cervical dislocation under anesthesia (secobarbital, 50 mg/kg, ip). Blood and kidney samples were collected; the blood (withdrawn from retro-orbital plexus) was subjected to centrifugation then serum was kept for biochemical measurements. The kidneys were isolated; the left kidney was directly mixed with ice-cold 10 mM phosphate buffer (pH 7.4) to yield a 10% (w/v) homogenate, which was then centrifuged for 10 min at 4 °C to obtain supernatant and the right one was kept in buffered formalin for histological evaluation.

### Estimation of serum parameters

In order to biochemically assess renal damage and function, serum levels of creatinine and urea were estimated using commercially available kits from Biodiagnostic Co. (Giza, Egypt) according to the attached procedures. Blood urea nitrogen (BUN) was deduced using the following formula: BUN (mg/dL) = urea/2.14 [12].

### Estimation of urine parameters

Urinary levels of creatinine, albumin and total protein were assessed using kits from Biodiagnostic Co. (Giza, Egypt) following the instructions provided.

### Assessment of oxidative stress

Following the method described by Okhawa, renal MDA content was determined as an index of lipid peroxidation [13]. Regarding reduced glutathione (GSH), its renal level was measured according to Ellman’s method [14]. Concerning superoxide dismutase (SOD), its activity was assessed according to Marklund method [15].

### Determination of inflammatory mediators

Renal levels of toll-like receptor 4 (TLR-4), nuclear factor kappa B (NF-κB) were determined using ELISA kits from Wuhan Fine Biotech Co., Ltd. (China). Renal levels of NOD-like receptor protein 3 (NLRP3), apoptosis associated speck-like protein (ASC), Caspase-1 and interleukin (IL)-1β were measured by means of ELISA kits from MyBioSource (USA), Abbexa LTD, BioVision (Switzerland) and BioLegend, Inc. (USA) respectively.

### Histopathology

The renal tissues preserved in buffered formalin were embedded in paraffin wax, trimmed into 4-µm-thick sections using a microtome, and then stained with hematoxylin and eosin (H&E) for histolopathological evaluation. The scoring procedure was carried out as previously mentioned [16]: the injury area is 0%, 0 points, normal; 1 point, mild; 25–50%, 2 points, moderate; the injury area is less than 25%, 1 point, 3 scores, serious; damage area >75%, 50–75%.

### Immunohistochemical analysis

Renal sections were utilized in accordance with the established protocols for IHC evaluation using a monoclonal mouse anti-Human COX-II antibody, Clone CX-294, Dako, Denmark (anti-COX-II) (1:100 dilution) [17]. Assessment of immunostaining was carried out by determining the percentage of positive areas using image J analysis software.

### Statistical analysis

Data are represented as mean ± standard error of the mean (S.E.M.). Tukey’s Kramer multiple comparisons test after one-way analysis of variance (ANOVA). The data were applied. Graphpad software Prism V 5 (Graphpad Software Inc., San Diego, CA, USA) was used to conduct statistical analyses. A p-value < 0.05 was considered significant.

## Results

### Effect of tiron (140 & 280 mg/kg) on general data

Table 1 showed that Diclo significantly increased kidney/body weight ratio (by 1.4-fold) and concurrently decreased final body weight (by 27.4%) and urine volume (by 86.4%) relative to the control group. Conversely, tiron in both doses significantly decreased kidney/body weight ratio (by 19.1% and 17.1%, respectively) and increased final body weight (by 1.2- and 1.3- fold, respectively) and urine volume (by 3- and 3.6- fold, respectively) relative to the Diclo group.

**Table 1:** Effect of tiron (140 & 280 mg/kg) on body weights, kidney to body weight ratio and urine volume

	Initial body weight (gm)	Final body weight (gm)	Kidney to body weight ratio (X10^− 3^)	Urine volume (ml)
Control	22.5 ± 0.4	24.63 ± 0.7	11.3 ± 0.18	3.7 ± 0.07
Diclo	22.8 ± 0.5	17.88 ± 0.65^$^	16.05 ± 0.59^$^	0.5 ± 0.04^$^
Tiron 1	22.5 ± 0.4	23 ± 0.7^#^	12.98 ± 0.34^$#^	1.5 ± 0.07^$#^
Tiron 2	22.2 ± 0.3	23.25 ± 0.7^#^	13.3 ± 0.27^$#^	1.8 ± 0.07^$#*^

Tiron (140 & 280 mg/kg/day) were orally administered for 7 days prior to Diclo administration. On day 8, body weights, volume of urine and kidney to body weight ratio were recorded. Values are presented as mean ± SEM (*n* = 8). $,#, * (*P* > 0.05) significance relative to Control, Diclo group, Tiron 1 respectively using one-way ANOVA followed by Tukey-Kramer multiple comparisons post hoc test

### Effect of tiron (140 & 280 mg/kg) on serum parameters

**Fig. 1:**
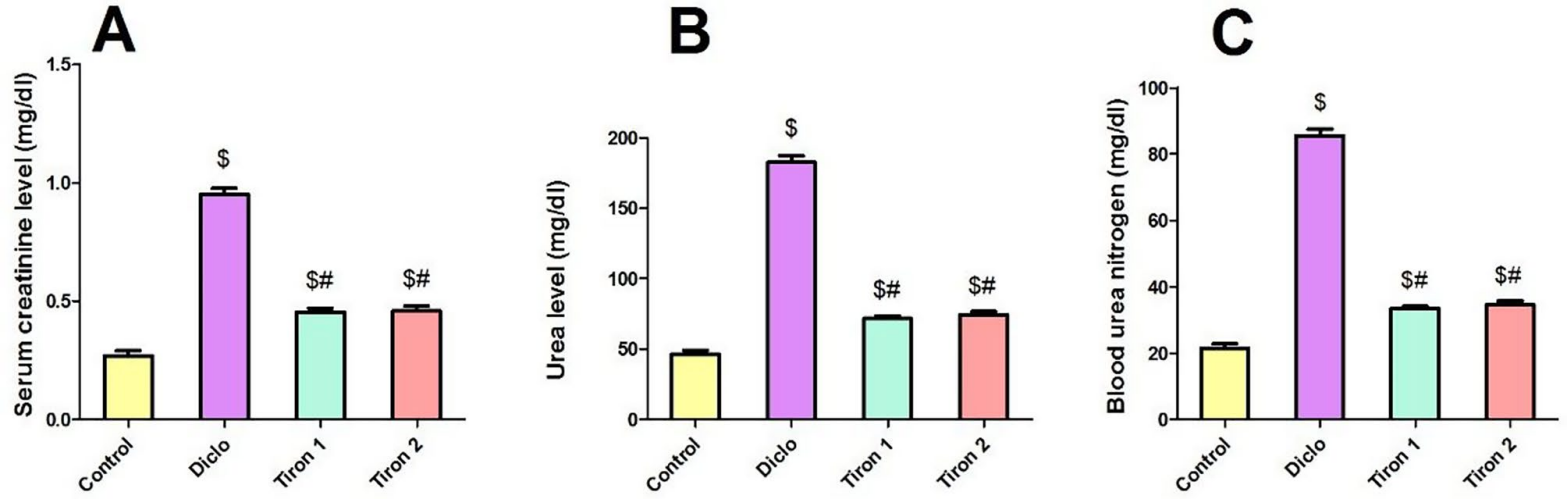
Effect of tiron (140 & 280 mg/kg/day, P.O.) on serum parameters. (**A**) Serum creatinine, (**B**) Urea, (**C**) Blood urea nitrogen. Data are presented as mean ± SEM (*n* = 8) using one-way ANOVA followed by Tukey-Kramer comparison post hoc test. $, #(*P* > 0.05) significance relative to Control, Diclo group, respectively

Administration of Diclo (300 mg/kg) resulted in renal damage in mice evidenced by significant rise in serum levels of creatinine, urea and BUN by 3.5-fold, 3.9-fold and 3.9-fold respectively relative to control group. Pretreatment with tiron (140 & 280 mg/kg) significantly decreased the serum levels of creatinine (52.6% & 51.5%, respectively), urea (60.7% & 59.4%, respectively) and BUN (60.9% &59.5%, respectively) relative to Diclo group (Fig. 1).

### Effect of tiron (140 & 280 mg/kg) on urine parameters

**Fig. 2:**
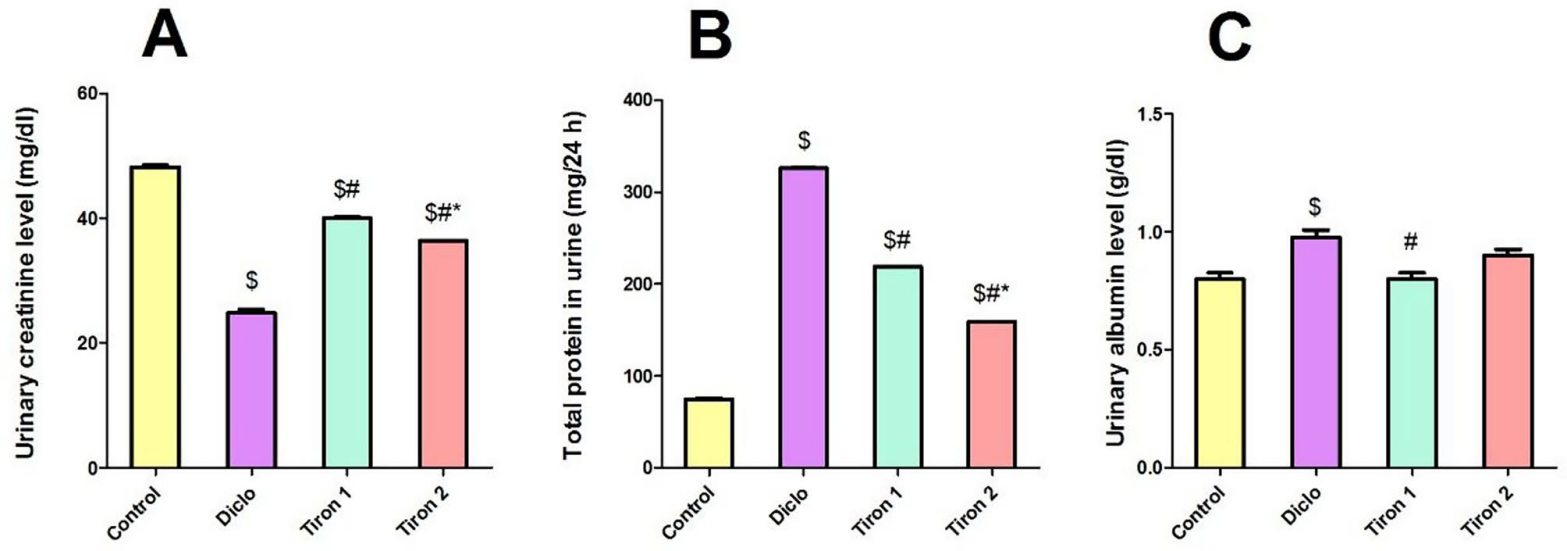
Effect of tiron (140 & 280 mg/kg) on urine parameters. (**A**) Urinary creatinine, (**B**) Total protein, (**C**) Albumin. Data are presented as mean ± SEM (*n* = 8) using one-way ANOVA followed by Tukey-Kramer comparison post hoc test. $, # (*P* > 0.05) significance relative to Control, Diclo group, respectively

Diclo induced a marked decrease in urinary levels of creatinine by 48.4% relative to the control group. Pretreatment with tiron (140 & 280 mg/kg) considerably increased the urinary levels of creatinine by 1.61-fold & 1.4- fold, respectively (Fig. 2A).

Concomitantly, Diclo substantially increased urinary levels of total protein and albumin by 4.3-fold and 1.2-fold respectively relative to the control group. However, pretreatment with tiron (140 & 280 mg/kg) reduced the urinary levels of total protein (by 32.8% & 51.2%, respectively) and albumin (by 17.9% and 7.6%, respectively) when compared to Diclo group (Fig. 2B and C).

### Effect of tiron (140 & 280 mg/kg) on renal oxidant/antioxidant status

Table 2 revealed that Diclo significantly elevated MDA content (by 3-fold) and simultaneously decreased renal GSH level (by 22.9%) and SOD activity (by 23.9%) relative to the control group. Conversely, tiron in both doses significantly improved the redox balance in the kidney tissues evidenced by the profound reduction in MDA content (by 40.2% & 43.5%, respectively) and meaningful increase in GSH level (by 1.28-fold and 1.43-fold, respectively) and SOD activity (by 1.24-fold & 1.25-fold) relative to Diclo group.

**Table 2:** Effect of tiron (140 & 280 mg/kg) on antioxidant status

	MDA (nmol/mg tissue)	GSH (nmol/mg tissue)	SOD (U/mg)
Control	5.87 ± 0.48	30.9 ± 2.2	57.1 ± 0.65
Diclo	17.9 ± 0.65^$^	23.8 ± 0.59^$^	44 ± 1.76^$^
Tiron 1	10.7 ± 0.62^$#^	30.5 ± 1.48^#^	54.6 ± 0.34^#^
Tiron 2	10.1 ± 0.62^$#^	34.2 ± 0.64^#^	55 ± 0.92^#^

Tiron (140 & 280 mg/kg/day) were orally administered for 7 days prior to Diclo administration. On day 8, kidney tissues were collected to assess oxidative stress biomarkers. Values are presented as mean ± SEM (*n* = 8). $, # (*P* > 0.05) significance relative to Control, Diclo group, respectively using one-way ANOVA followed by Tukey-Kramer multiple comparisons post hoc test

Diclo, diclofenac; GSH, reduced glutathione; MDA, malondialdehyde; SOD, superoxide dismutase

### Effect of tiron (140 & 280 mg/kg) on renal TLR4 & NF-κBp65 levels

**Fig. 3:**
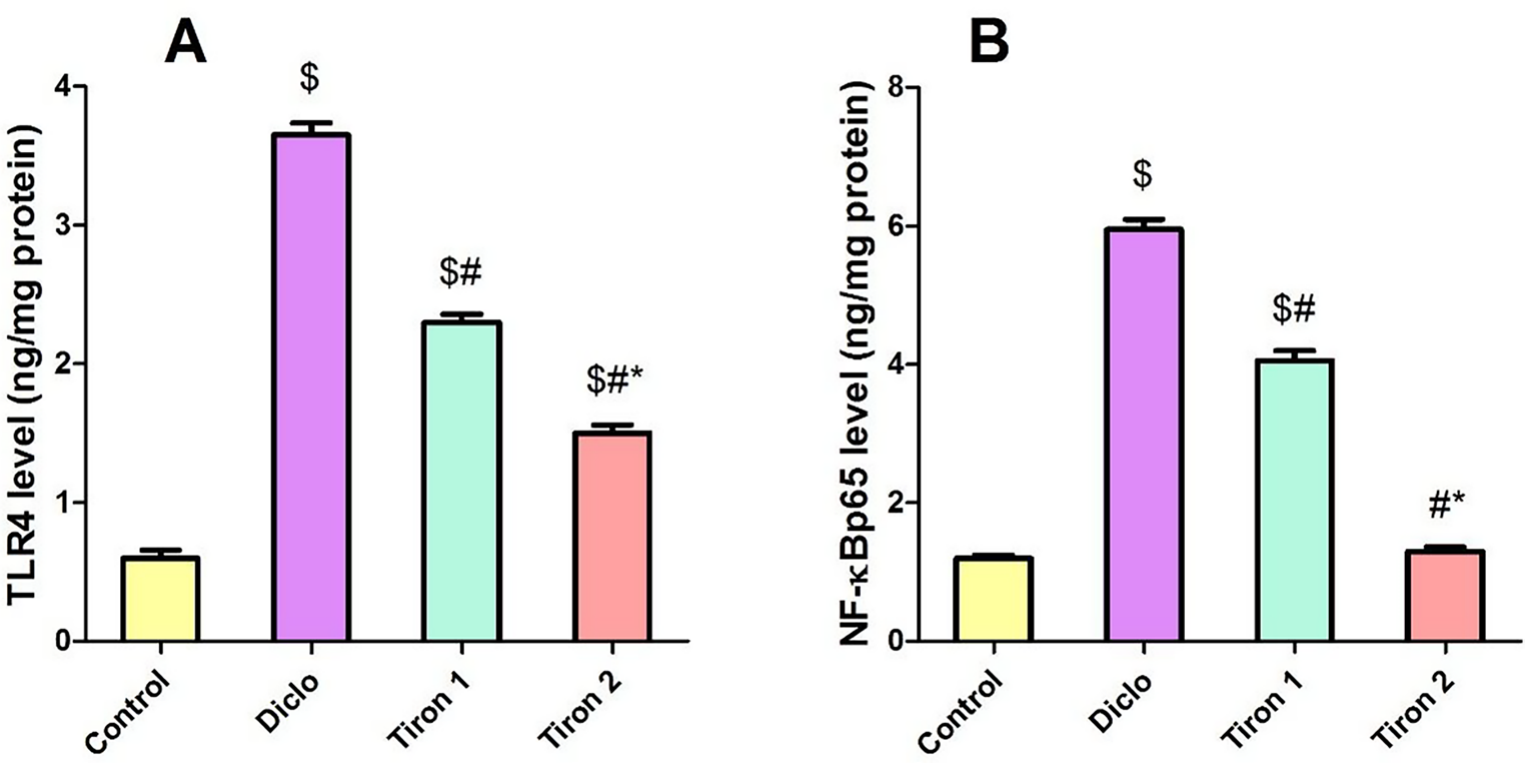
Effect of tiron (140 & 280 mg/kg) on renal TLR4 & NF-κBp65 levels. (**A**) TLR4, (**B**) NF-κBp65. Data are presented as mean ± SEM (*n* = 8) using one-way ANOVA followed by Tukey-Kramer comparison post hoc test. $, #, * (*P* > 0.05) significance relative to Control, Diclo group, Tiron 1, respectively

In order to assess the anti-inflammatory aspects of tiron, both levels of renal TLR4 & NF-κBp65 were determined. Diclo markedly increased renal levels of TLR4 & NF-κBp65 by 6-fold and 4.9-fold respectively relative to the control group. Tiron in both doses produced a profound decline in renal levels of TLR4 (by 36.9% and 58.9%, respectively) and NF-κBp65 (by 31.9% and 78.1%, respectively) relative to Diclo group (Fig. 3).

### Effect of tiron (140 & 280 mg/kg) on NLRP3 pathway

**Fig. 4:**
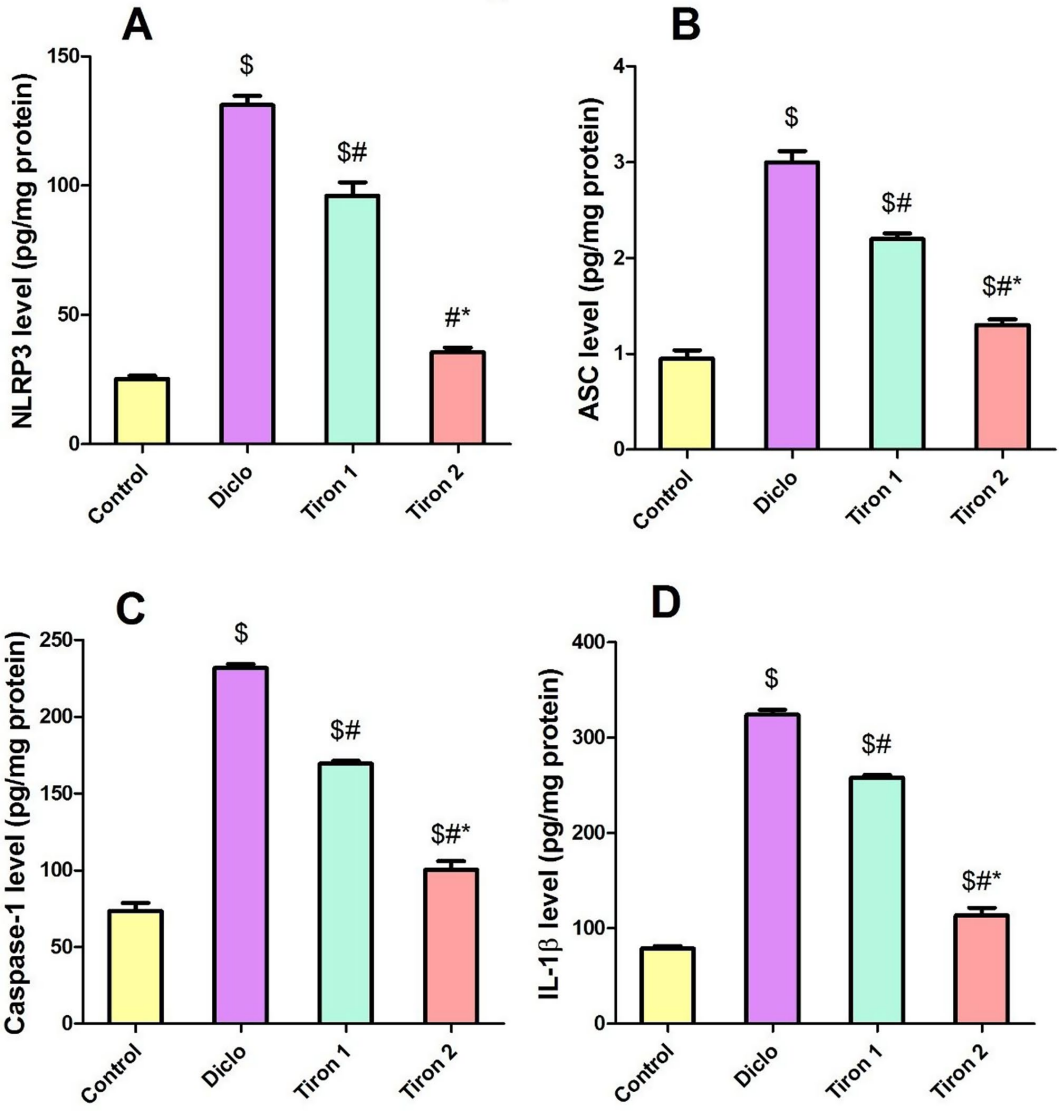
Effect of tiron (140 & 280 mg/kg) on NLRP3 pathway. (**A**) NLRP3, (**B**) ASC, (**C**) Caspase-1, (**D**) IL-1β. Data are presented as mean ± SEM (*n* = 8) using one-way ANOVA followed by Tukey-Kramer comparison post hoc test. $, #, * (*P* > 0.05) significance relative to Control, Diclo group, Tiron 1, respectively

To further confirm the anti-inflammatory effects of tiron, the signaling pathway of NLRP3 inflammosome has been assessed. Diclo substantially elevated renal levels of NLRP3, ASC, Caspase1 and IL-1β by 5.1-fold, 3.1-fold, 3.1-fold and 4.1-fold respectively when compared to the control group. Pretreatment with tiron in both doses resulted in a considerable reduction in renal levels of NLRP3 (by 26.6% & 72.8%, respectively), ASC (26.6% & 56.6%, respectively), Caspase-1 (26.8% & 56.6%, respectively) and IL-1β (20.3% & 64.9%, respectively) when compared to Diclo group (Fig. 4).

### Effect of tiron (140 & 280 mg/kg) on histopathological examination of renal tissues

**Fig. 5:**
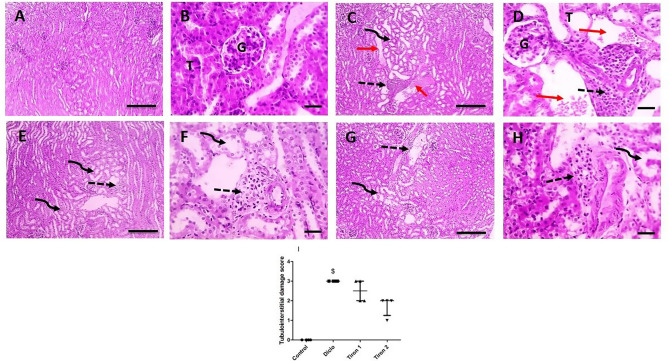
Effect of tiron (140 & 280 mg/kg) on histopathological examination of renal tissues. Microscopic pictures of H&E stained renal sections show normal structure of cortical glomeruli (**G**) and tubules in the control group (**A**&**B**). Renal sections from Diclo group (**C**&**D**) received show focal area of tubular dilation (curved arrow), congestion (red arrows), focal interstitial lymphocytic infiltration (dashed arrow). Renal sections from Tiron 1 group (**E**&**F**) show focal area of tubular dilation (dashed arrow), focal few interstitial lymphocytic infiltration (curved arrow). Renal sections from Tiron 2 group (**G**&**H**) show decrease in tubular dilation (curved arrow) with presence of very few interstitial lymphocytic infiltration (dashed arrow). Low magnification power X: 100 bar 100 (**A**, **C**, **E** & **G**). High magnification power X: 400 bar X: 50 (**B**, **D**, **F** & **H**). (**I**) Semiquantitative scoring of tubulointerstitial damage within different treated groups. Kruskal-Wallis test followed by Dunn’s post-hoc test were performed. $ Significantly different from control group (*n* = 4)

Figure 5 represents the histopathological alterations in renal tissues. Renal sections from the control group appears normal (Fig. 5A and B). Renal sections from Diclo group shows massive areas of congestion and focal interstitial lymphocytic infiltration (Fig. 5C and D). Sections of this group also affirm glomerular affection through appearance of focal area of tubular dilation with presence of eosinophilic proteinaceous material in renal tubules. Renal sections from Tiron 1 group show few interstitial lymphocytic infiltration and mild glomerular affection in the form of tubular dilation (Fig. 5E and F). While renal sections from Tiron 2 group (Fig. 5G and H) show very few interstitial lymphocytic infiltration with significant decrease in tubular dilation compared to Diclo group.

### Effect of tiron (140 & 280 mg/kg) on immunohistochemical examination of renal tissues

**Fig. 6:**
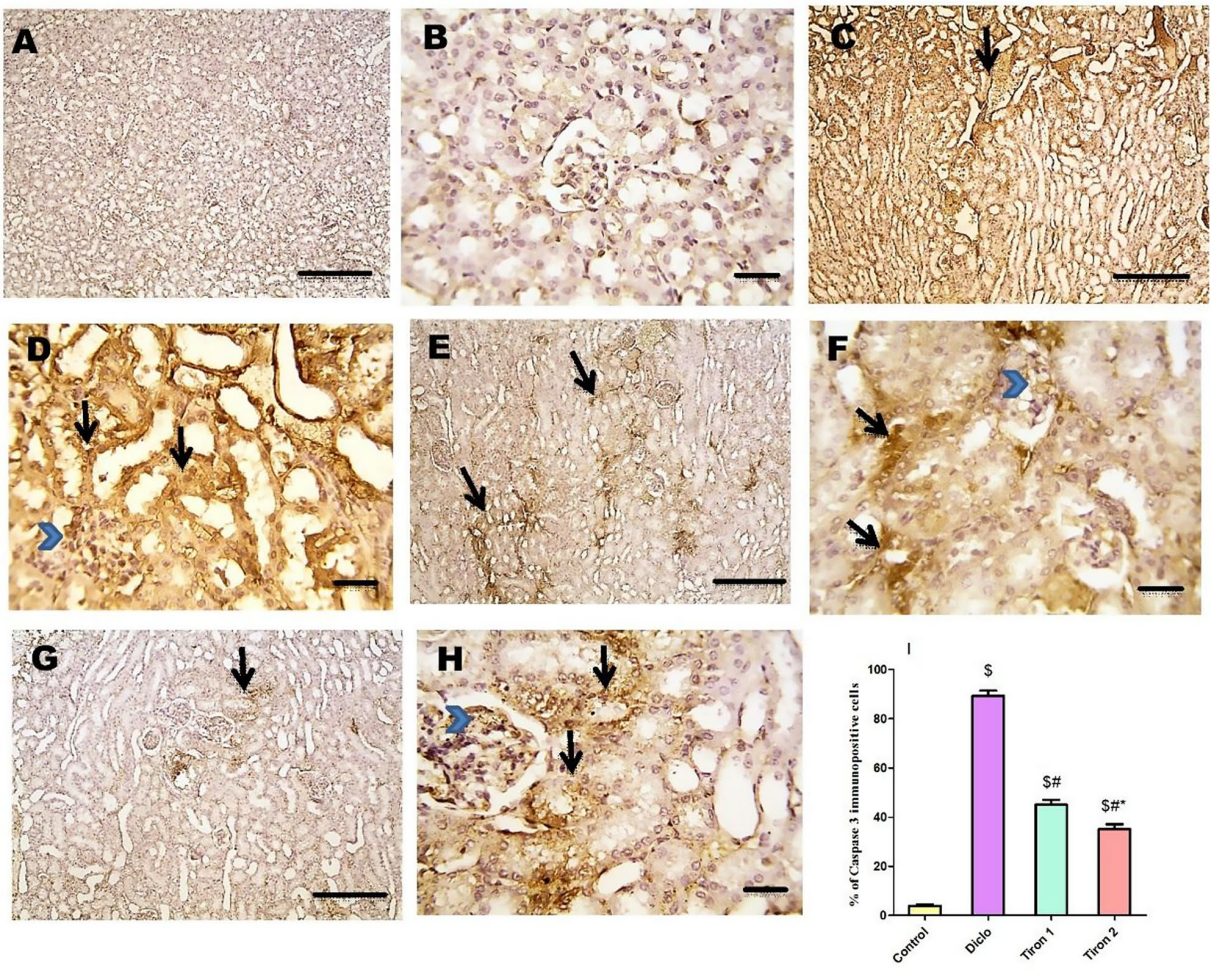
Effect of tiron (140 & 280 mg/kg) on immunohistochemical examination of renal tissues. Microscopic pictures of immunostained renal sections from control group (**A**&**B**) against COX-II show absent expression in tubules & glomeruli. Microscopic pictures of immunostained renal sections from Diclo group against COX-II show strong positive brown expression in tubules (arrows) &glomeruli (arrowheads) (**C**&**D**). Renal sections from Tiron 1 group show moderate positive brown expression against Cox-II that appears in some tubules (arrows) & glomeruli (arrowheads) (**E**&**F**). Renal sections from Tiron 2 group show mild positive brown expression against COX-II that appears in few tubules (arrows) & glomeruli (arrowheads) (**G**&**H**). IHC counterstained with Mayer’s hematoxylin. Low magnification X:100 bar 100 (**A**, **C**, **E** & **G**). High magnification power X: 400 bar X: 50 (**B**, **D**, **F** & **H**). (**I**) Semi-quantitative analysis of COX-II immunostaining results in renal tissues expressed as % of COX-II immunopositive cells. Data are presented as mean ± SEM (*n* = 4) using one-way ANOVA followed by Tukey-Kramer comparison post hoc test. $, #, * (*P* > 0.05) significance relative to Control, Diclo group, Tiron 1, respectively

Microscopic pictures of immunostained renal sections from control group against COX-II show absent expression in tubules & glomeruli (Fig. 6A and B). Microscopic pictures of immunostained renal sections from Diclo group against Cox-II show strong positive brown expression in tubules &glomeruli (Fig. 6C and D). Renal sections from Tiron 1 group show moderate positive brown expression against COX-II that appears in some tubules & glomeruli (Fig. 6E and F). Renal sections from Tiron 2 group show mild positive brown expression against COX-II that appears in few tubules & glomeruli (Fig. 6G and H). IHC counterstained with Mayer’s hematoxylin. Low magnification X:100 bar 100 (A, C, E & G). High magnification power X: 400 bar X: 50 (B, D, F & H).

## Discussion

This study was designated to explore the probable renoprotective impact of tiron against diclofenac-induced acute kidney injury (AKI). Diclofenac is a well-established model used to induce acute nephrotoxicity experimentally [4]. Our results showed that single dose of diclofenac (300 mg/kg) induced nephrotoxicity evidenced by increased levels of serum creatinine, urea and BUN in addition to increased proteinuria and albuminuria concomitant with marked decrease in urinary creatinine level when compared to the control group. These biochemical results were further confirmed by hisopathological analysis which revealed congestion and focal interstitial lymphocytic infiltration in Diclo group. These findings agreed with earlier studies that reported diclofenac-induced nephrotoxicity [6, 18].

On the other hand, pretreatment with tiron showed marked improvement in both renal function and structure as manifested by marked reduction in serum creatinine, urea and BUN besides decreased proteinuria and albuminuria concomitant with profound increase in urinary creatinine level when compared to Diclo group. In addition to the improvement in histopathological alterations induced by diclofenac. These observations are in harmony with an earlier research which showed that tiron attenuated the nephrotoxicity induced by titanium dioxide nanoparticles [19].

Oxidative stress has been demonstrated to be associated with the developmentof acute nephrotoxicity [20]. More precisely, the oxidative pathway is significantly activated in diclofenac-induced acute nephrotoxicity. Actually, diclofenac causes oxidative stress via altering kidney mitochondrial complex I, which lowers the production of ATP, according to in vitro evidence [21]. Our observations showed imbalance in oxidant/antioxidant status reflected by increased MDA content and lower level of GSH and SOD activity after diclofenac administration. On the other hand, pretreatment with tiron effectively restored oxidant/antioxidant balance as evidenced by reduced MDA content and elevated GSH level and SOD activity. These findings are in harmony with earlier studies which reported the anti-oxidant properties of tiron in models of breast cancer [7] and Parkinsonism [10].

Oxidant/antioxidant imbalance prompts the galvanization of inflammatory pathways mainly via stimulation of the transcription factor NF-κB pathway which in turn increases the expression of several inflammatory mediators, comprising NLRP3 inflammasome [22]. Several AKI models are mediated by the NLRP3 inflammasome, which can be assembled in response to a variety of documented stimuli, including ROS buildup and activation of the NF-κB pathway [23]. The NLRP3 protein, ASC, and pro-caspase-1 make up the NLRP3 inflammasome. The activation of NLRP3 causes caspase-1 to be cleaved, which in turn stimulates the production of cytokines and IL-1β. It has been reported that the raised expression levels of NLRP3, ASC, and cleaved caspase-1, along with the elevated levels of IL-1β in kidney tissues, demonstrated that diclofenac activated the assembly of the NLRP3 inflammasome [3].

Toll like receptors (TLRs) are a group of receptors that are implicated in innate immunity besides inflammation. It have been documented that TLR4 is linked to the pathophysiology of both acute and chronic kidney diseases. TLR4 triggers the cytoplasmic NLRP3, which activates pro-caspase-1 to active Caspase-1 which in turn activates proactive IL-1β/18 [22]. Also, it is known that stimulation of TLR4 signaling pathway initiates a cascade of events that include NF-κBp65 translocation to the nucleus, which leads to the production of inflammatory cytokines which affect both survival and death of cells [24]. Moreover, oxidative stress contributes to NF-κB activation, which in turn prompts the release of the consequent inflammatory cytokines [25]. NF-κB is a master switch that controls the transcription of inflammatory mediators like COX-II [26].

Correspondingly, our data revealed that diclofenac significantly elevated renal levels of TLR4, NF-κBp65, NLRP3, ASC, Caspase-1 and IL-1β in addition to increased renal expression of COXII relative to the control group. These results are in harmony with earlier researches which reported that diclofenac induces inflammation in renal tissues [6, 27]. The observed rise in oxidative stress production and NF-κB pathway activation corroborated these results, indicating the involvement of the NLRP3 inflammasome and associated regulatory components in diclofenac-induced AKI. On contrast, tiron pretreatment markedly suppressed the activation of both TLR4/NF-κB and NLRP3 signaling pathways in the kidney concomitant with inhibition of renal expression of COXII. This implies that the anti-inflammatory properties of tiron may be largely responsible for its protective effect against diclofenac-induced nephrotoxicity. These findings supported the notion that tiron has an anti-inflammatory effect in many experimental models of inflammation by inhibiting the NLRP3 inflammasome response and down regulating the activation of the TLR4/NF-κB signaling pathways [9, 28].

## Conclusion

Together, tiron could confer protection against diclofenac-induced nephrotoxicity owing to its antioxidants and anti-inflammatory properties. This protection was mediated mainly through targeting TLR4/NF-κB and NLRP3 inflammatory pathways. Tiron is therefore a promising cadidate that could be used to prevent AKI that is linked to NSAID use. To guarantee this advantageous renoprotective impact, more clinical research is necessary.

## Data Availability

The datasets used and/or analyzed during the current study are available from the corresponding author on reasonable request.

## References

[1] Hosohata K. Role of oxidative stress in drug-induced kidney injury. Int J Mol Sci. 2016;17(11). 10.3390/ijms17111826 PMC5133827 27809280

[2] Kim SY, Moon A. Drug-induced nephrotoxicity and its biomarkers. Biomol Ther (Seoul). 2012;20(3):268–72. 24130922 10.4062/biomolther.2012.20.3.268 PMC3794522

[3] El-Maadawy WH, et al. 6-Paradol alleviates Diclofenac-induced acute kidney injury via autophagy enhancement-mediated by AMPK/AKT/mTOR and NLRP3 inflammasome pathways. Environ Toxicol Pharmacol. 2022;91:103817. 35091105 10.1016/j.etap.2022.103817

[4] Alorabi M, et al. Pentoxifylline and Berberine mitigate diclofenac-induced acute nephrotoxicity in male rats via modulation of inflammation and oxidative stress. Biomed Pharmacother. 2022;152:113225. 35671584 10.1016/j.biopha.2022.113225

[5] Gómez-Oliván LM, et al. Genotoxic response and oxidative stress induced by diclofenac, ibuprofen and Naproxen in daphnia magna. Drug Chem Toxicol. 2014;37(4):391–9. 24393029 10.3109/01480545.2013.870191

[6] Fattori V, et al. Vinpocetine reduces diclofenac-induced acute kidney injury through Inhibition of oxidative stress, apoptosis, cytokine production, and NF-κB activation in mice. Pharmacol Res. 2017;120:10–22. 28315429 10.1016/j.phrs.2016.12.039

[7] Abouelezz HM, et al. Tiron enhances the anti-cancer activity of doxorubicin in DMBA-induced breast cancer: role of Notch signaling/apoptosis/autophagy/oxidative stress. Food Chem Toxicol. 2024;193:114968. 39214269 10.1016/j.fct.2024.114968

[8] Oyewole AO, Birch-Machin MA. Mitochondria-targeted antioxidants. Faseb J. 2015;29(12):4766–71. 26253366 10.1096/fj.15-275404

[9] Abdelrahaman D, et al. Suppression of NLRP3 inflammasome orchestrates the protective efficacy of Tiron against isoprenaline-induced myocardial injury. Front Pharmacol. 2024;15:1379908. 39211776 10.3389/fphar.2024.1379908 PMC11358555

[10] Mohamed SA, El-Kashef DH, Nader MA. Tiron alleviates MPTP-induced parkinsonism in mice via activation of Keap-1/Nrf2 pathway. J Biochem Mol Toxicol. 2021;35(3):e22685. 33368846 10.1002/jbt.22685

[11] Hickey EJ, et al. Diclofenac induced in vivo nephrotoxicity May involve oxidative stress-mediated massive genomic DNA fragmentation and apoptotic cell death. Free Radic Biol Med. 2001;31(2):139–52. 11440826 10.1016/s0891-5849(01)00560-3

[12] Samaha MM, et al. Diacerein mitigates adenine-induced chronic kidney disease in rats: focus on TLR4/MYD88/TRAF6/NF-κB pathway. Life Sci. 2023;331:122080. 37690574 10.1016/j.lfs.2023.122080

[13] Ohkawa H, Ohishi N, Yagi K. Assay for lipid peroxides in animal tissues by thiobarbituric acid reaction. Anal Biochem. 1979;95(2):351–8. 36810 10.1016/0003-2697(79)90738-3

[14] Ellman GL. Tissue sulfhydryl groups. Arch Biochem Biophys. 1959;82(1):70–7. 13650640 10.1016/0003-9861(59)90090-6

[15] Marklund S, Marklund G. Involvement of the superoxide anion radical in the autoxidation of pyrogallol and a convenient assay for superoxide dismutase. Eur J Biochem. 1974;47(3):469–74. 4215654 10.1111/j.1432-1033.1974.tb03714.x

[16] Ezzat DM, Soliman AM, El-Kashef DH. Nicorandil mitigates folic acid-induced nephrotoxicity in mice: role of iNOS and eNOS. J Biochem Mol Toxicol. 2021;35(4):e22692. 10.1002/jbt.22692 33404076

[17] Abdo W, et al. Combined effects of organochlorine pesticides heptachlor and hexachlorobenzene on the promotion stage of hepatocarcinogenesis in rats. Food Chem Toxicol. 2013;55:578–85. 23402856 10.1016/j.fct.2013.01.035

[18] Mansoure AN, Elshal M, Helal MG. Inhibitory effect of diacerein on diclofenac-induced acute nephrotoxicity in rats via modulating SIRT1/HIF-1α/NF-κB and SIRT1/p53 regulatory axes. Int Immunopharmacol. 2024;131:111776. 38471363 10.1016/j.intimp.2024.111776

[19] Morgan A, et al. Tiron ameliorates oxidative stress and inflammation in titanium dioxide nanoparticles induced nephrotoxicity of male rats. Biomed Pharmacother. 2017;93:779–87. 28709131 10.1016/j.biopha.2017.07.006

[20] Rasheed HA, Al-Kuraishy HM, Al-Gareeb AI. Rosuvastatin attenuates acute nephrotoxicity through modulation of oxidative stress in Sprague Dawley rats. J Pak Med Assoc. 2019;69(Suppl 3):S98–102. 31603887

[21] Ng LE, et al. Action of diclofenac on kidney mitochondria and cells. Biochem Biophys Res Commun. 2006;348(2):494–500. 16890207 10.1016/j.bbrc.2006.07.089

[22] Mansoure AN, Elshal M, Helal MG. Renoprotective effect of Diacetylrhein on diclofenac-induced acute kidney injury in rats via modulating Nrf2/NF-κB/NLRP3/GSDMD signaling pathways. Food Chem Toxicol. 2024;187:114637. 38582345 10.1016/j.fct.2024.114637

[23] Chen CY, et al. Ugonin U stimulates NLRP3 inflammasome activation and enhances inflammasome-mediated pathogen clearance. Redox Biol. 2017;11:263–74. 28012441 10.1016/j.redox.2016.12.018 PMC5198739

[24] El-Kashef DH, Serrya MS. Sitagliptin ameliorates thioacetamide-induced acute liver injury via modulating TLR4/NF-KB signaling pathway in mice. Life Sci. 2019;228:266–73. 31077717 10.1016/j.lfs.2019.05.019

[25] El-Kashef DH. Nicorandil ameliorates pulmonary inflammation and fibrosis in a rat model of silicosis. Int Immunopharmacol. 2018;64:289–97. 30223191 10.1016/j.intimp.2018.09.017

[26] Kumar J, Haldar C. Melatonin ameliorates LPS-induced testicular nitro-oxidative stress (iNOS/TNFα) and inflammation (NF-kB/COX-2) via modulation of SIRT-1. Reprod Sci. 2021;28(12):3417–30. 10.1007/s43032-021-00597-0 33929710

[27] Shafeek F, et al. Gum acacia mitigates diclofenac nephrotoxicity by targeting monocyte chemoattractant protein-1, complement receptor-1 and pro-apoptotic pathways. Food Chem Toxicol. 2019;129:162–8. 31042592 10.1016/j.fct.2019.04.050

[28] El-Sherbeeny NA, Hassan ZA, Ateyya H. Tiron ameliorates oxidative stress and inflammation in a murine model of airway remodeling. Int Immunopharmacol. 2016;39:172–80. 27485290 10.1016/j.intimp.2016.07.025

